# Assessment of skin-to-skin contact (SSC) during the postpartum stay and its determinant factors among mothers at public health institutions in Ethiopia

**DOI:** 10.1186/s13104-019-4176-5

**Published:** 2019-03-14

**Authors:** Asres Bedaso, Emnet Kebede, Tariku Adamu

**Affiliations:** 1 School of Nursing, College of Medicine and Health Sciences, Hawassa University, Hawassa, SNNPR Ethiopia; 2 Department of Midwifery, College of Medicine and Health Sciences, Hawassa University, Hawassa, SNNPR Ethiopia; 3 Department of Midwifery, College of Medicine and Health Science, Assosa University, Assosa, Ethiopia

**Keywords:** Skin-to-skin contact, Determinants, Mothers, Postpartum stay, Ethiopia

## Abstract

**Objective:**

The study aimed at assessing skin-to-skin contact (SSC) during the postpartum stay and its determinant factors among mothers at public health institutions in Ethiopia.

**Result:**

A total of 384 mothers-indexed newborns admitted in postnatal wards were interviewed. The prevalence of mothers’ SSC practice to their newborns with in the first 1 h during the postpartum stay was 28.1%. Mothers education (AOR = 18.23 [95% CI 5.26, 63.52]), and number of ANC visits (AOR = 8.55 [95% CI 1.05, 69.54]) were independently associated with SSC practice of mothers to their infants.

**Electronic supplementary material:**

The online version of this article (10.1186/s13104-019-4176-5) contains supplementary material, which is available to authorized users.

## Introduction

Early skin-to-skin contact (SSC) is defined as placing the naked baby, covered across the back with a warm blanket, prone on the mother’s bare chest [1]. Skin-to-skin contact between mother and baby after birth reduces crying, improves mother-baby interaction, keeps the baby warmer, and helps women breastfeed successfully. Long-lasting SSC throughout the postpartum period is important to decrease the frequency of formula supplementation for breast feeding and it will improve acquisition of the maternal role [1, 2].

Regardless of the importance of early mother–newborn SSC immediately after birth, it has not been universally implemented as routine care for healthy term neonates, and separation of mothers and infants is common in many health facilities. [3].

According to UNICEF 2.9 million babies die each year within 28 days and the first 24 h after birth are the most risky period both for the child and the mother. In Ethiopia, an approximate 84,400 new born die within the first month of birth, according to the Lancet Series of Every New-born [4].

Some low and middle-income countries are making significant improvement by providing training for midwifery and nurses professionals to address the poorest families with good quality care at birth, particularly for small or ill new-born. Ethiopia is dedicated in minimizing the under-five mortality rate to 68 deaths per 1000 live births by 2015 [5]. Nevertheless, as in other developing countries, the reduction is mainly a result of fewer deaths in children 1 to 59 months old, while neonatal (first 28 days of life) mortality has shown more modest reaching 37 deaths per 1000 live births in 2011 according to EDHS [6].

There has been many research surrounding early SSC hours after birth, but far less in assessing the practice after the first hours and throughout the hospital stay during postpartum period. The effect of early SSC shortly after birth is so influential that it appears to surely contribute for exclusive breast feeding and the quality of mother–baby interactions [7].

Therefore, identification of perceived barriers held by mothers is vital in order to conduct interventions to facilitate early SSC. It is possible that increasing SSC in the postpartum period may reduce the frequency of formula supplementation during their hospital admission, a practice that is strongly linked with decreased breast feeding duration [8–10].

For mothers, SSC reduced postpartum bleeding, decreased the time it took to expel the placenta, increased oxytocin levels, and decreased cortisol levels, resulting in happier childbirth experiences, improved bonding with newborns, and increased confidence in their ability to care for the newborn [11–13]. Also, it is found that a dose-dependent correlation between the duration of SSC with full-term newborns initiated within the first 3 h of child-birth and the establishment of exclusive breastfeeding [14].

The importance of SSC continues beyond the first hour. The longer and more often mothers and babies are on SSC in the hours and days after the birth, the greater the benefit to get [15]. A recent study reported that Skin to skin contact in the postpartum period may also minimize the occurrence of postpartum depression, a condition that adversely affects mother/baby bonding, [16]. Also, SSC immediately after birth, which lasts for at least an hour has positive effects like maintaining body temperature normal, maintains heart rate, respiratory rate and blood pressure normal, and more likely to breastfeed exclusively and breastfeed longer [17].

Therefore the study aimed to assess SSC during the Postpartum Stay and its determinant factors among mothers at public health institutions in Ethiopia.

## Main text

### Methods

#### Study design, area and period

Institutional based cross-sectional study design was employed among mothers who gave live births at public health institutions in four selected towns, Ethiopia. This study was carried out in Addis Ababa (Central Ethiopia), Bahir Dar (Amhara National Regional State), Shashemene (Oromia Regional State) and Pawi (Benshangule Gumuz National Regional State). This study comprised Addis Ababa at St. Paul’s Hospital Medical Millennium College (SPHMMC). There are two referral hospitals and 38 health centers which provide maternal, newborn and child health (MNCH) and delivery services to their catchment areas in all the selected study areas. The study was conducted from January 2016 to May 2017, in the four selected cities public health institutions of post-natal units.

#### Source and study population

Source population for this study was all mothers who gave a live birth at public health institutions. Study population were all mothers who gave live births and admitted at post-natal wards for at least for the first 6 h in the selected public health facilities during the study period and fulfill the inclusion criterion.

#### Inclusion and exclusion criteria

Mothers who gave singleton live births vaginally and admitted for at least the first 6 h post-natal period during data collection period were included in the study. Mothers who developed serious postpartum complications/unconscious were excluded from the study.

#### Sample size determination and sampling procedure

Single Population proportion formula was used to calculate the sample size considering the following assumptions: 95% confidence level, margin of error (0.05), 50% prevalence of early SSC since there is no previous research conducted on early SSC practice in Ethiopia. Considering 10% non response rate the final estimated sample size was 384. Simple random sampling technique was employed to select public health institutions in the four selected study areas. A mother who gave live births and admitted in post-natal units during the first 6 h was taken proportionally from each selected health facilities post-natal wards.

Four of the health facilities were selected using simple random sampling technique. Study subjects were obtained proportionally to the client flow from each post-natal unit and all mothers who gave live births in the selected public health facilities during the data collection period was interviewed consecutively with convenience to respond until the predetermined sample size was obtained (Additional file 1).

#### Instruments

A structured interview questionnaire was adopted from different literatures and used to explore the objective of the study, which was prepared in English and translated into Amharic (local language).

#### Data collection technique

Data collection was conducted by trained data collectors from January 2016 to May 2017 in each selected public health facilities during working hours. After getting written consent from each mother, interview was conducted by trained Midwives in each post-natal units of the study site. Pre-test was done on 5% of the study participants. Additional modifications and adjustments of the questionnaire were made based on the results of the pre-test.

#### Study variables

##### Dependent variable

SSC practice status of mothers during postpartum stay.

##### Independent variables

Socio-demographic characteristics of mothers: age, marital status, educational status, residence. Newborn characteristics: sex, weight and prematurity. Obstetric related characteristics: type of pregnancy, ANC follow-up, parity, type of delivery, birth attendant profession and type of health institution.

#### Data analysis

The data was entered using Epi-Info version 3.5.1, and exported to SPSS version 20 for analysis. SSC practice status was assessed by using self-report method and actual observation during data collection time. The association between variables to the outcome variable was measured by OR with 95% confidence interval. The strength of statistical association was measured by adjusted odds ratios and 95% confidence intervals. Statistical significance was declared at P < 0.05 and variables which showed statistical significant association (P < 0.2) during the bi-variate logistic regression was included in the final model.

### Result

#### Mothers’ and newborn’s socio-demographic characteristics

A total of 384 mothers of newborn were interviewed with 100% response rate. The mean age mothers’ is 26.47 years (SD ± 5.04) and 139 (36.2%) mothers were between the ages of 25–29 years and 72.9% of study participants were from urban areas (Table 1).

**Table 1:** Mothers’ with their indexed babies’ characteristics of SSC practice status during postpartum stay at public health institutions in four selected cities post-natal units, Ethiopia, 2016/17

Variables	Frequency	Percent	SSC status (N = 384)			
			Yes (n = 108)		No (n = 276)	
			n	%	n	%
Age						
16–20	25	6.5	6	24.0	19	76.0
21–24	105	27.3	37	35.2	68	64.8
25–29	139	36.2	40	22.8	99	71.2
30–34	79	20.6	18	22.8	61	77.2
> 35	36	9.4	7	19.4	29	80.6
Residence						
Urban	280	72.9	100	35.7	180	64.3
Rural	104	27.1	8	7.7	96	92.3
Religion						
Orthodox	249	64.8	76	30.5	173	69.5
Muslim	73	19.0	19	26.0	54	74.0
Protestant	49	12.8	11	22.4	38	77.6
Catholic	13	3.4	2	15.4	11	84.6
Ethnicity						
Amhara	228	59.4	74	32.5	154	67.5
Tigray	40	10.4	8	20.0	32	80.0
Oromo	116	30.2	26	22.4	90	77.6
Marital status						
Single	32	8.3	5	15.6	27	84.4
Married	333	86.7	100	30.0	233	70.0
Divorced	19	4.9	3	15.8	16	84.2
Educational status						
Unable to read and write	73	19.0	4	5.5	69	94.5
Primary (1–8)	119	31.0	25	21.0	84	79.0
Secondary (9–12)	113	29.4	45	39.8	68	60.2
Tertiary (Diploma and above)	79	20.6	34	43.0	45	57.0
Occupation						
Housewife	261	68.0	73	28.0	188	72.0
Employed	90	23.4	32	35.6	58	64.4
Unemployed	33	8.6	3	9.1	30	90.9
Newborns weight (in grams)						
≥ 4000	10	2.6	4	40.0	6	60.0
2500–3999	346	90.1	90	26.0	256	74.0
1500–2499	27	7.0	13	48.1	14	51.9
1000–1499	1	0.3	1	100.0	0	0.0
Sex						
Male	203	52.9	54	26.6	149	73.4
Female	181	47.1	54	29.8	127	70.2

#### Obstetrics Characteristics of Mothers

From the total study participants 315 (82%) of the pregnancy was unwanted and 348 (90.6%) have ANC follow up at health facility (Table 2).

**Table 2:** Obstetric characteristics of mothers indexed infants on SSC practice status during postpartum stay at public health institutions in four selected cities post-natal units, Ethiopia, 2016/17

Variables	Frequency	Percent	SSC status (N = 384)			
			Yes (n = 108)		No (n = 276)	
			n	%	n	%
Type of current pregnancy						
Wanted/planned	315	82.0	99	31.4	216	68.6
Unwanted/unplanned	69	18.0	9	13.0	60	87.0
ANC follow up						
Yes	348	90.6	107	30.7	241	69.3
No	36	9.4	1	2.8	35	97.2
Number of ANC visits						
One	17	4.4	1	5.9	16	94.1
Two	32	8.3	7	21.9	25	78.1
Three	67	17.4	15	22.4	52	77.6
Four	232	60.4	84	36.2	148	63.8
GA (gestational age)						
< 37 weeks	9	2.3	7	77.8	2	22.2
37–42 weeks	375	97.7	101	26.9	274	73.1
Parity						
Primi para	139	36.2	47	33.8	92	66.2
Multi para	245	63.8	61	24.9	184	75.1
Current birth attendant						
Midwife	274	71.4	88	32.1	186	67.9
Nurse	30	7.8	6	20.0	24	80.0
Doctor	25	6.5	6	24.0	19	76.0
Health officer	55	14.3	8	14.5	47	85.5
Mode of delivery						
Normal SVD	292	76.0	103	35.3	189	64.7
Assisted delivery	66	17.2	5	7.6	61	92.4
Abdominally (C/S)	26	6.8	0	0.0	26	100.0
Time after delivery						
< 6 h	295	76.8	77	26.1	218	73.9
6–12 h	50	13.0	16	32.0	34	68.0
> 12 h	39	10.2	15	38.5	24	61.5

#### Prevalence of mothers SSC practice

All (384) mothers were observed to look whether their infants attached with their body (skin), and about 154 (40.1%) of newborns were kept skin-to-skin with their mothers. Mothers were asked to indicate whether they provide SSC to their newborns during their postpartum stay for at least 1 h, and only 108 (28.1%) of mothers have practiced (Additional file 2).

#### Benefits of SSC

Among mothers who were asked about the advantages of SSC; 214 (55.7%) of mothers have responded as they know the purpose of SSC, and 170 (44.3%) of mothers didn’t know. Among mothers who know SSC importance 88 (22.9%) of them replied that SSC helps both to prevent hypothermia and facilitates EBF; 73 (19%) knew as it prevents hypothermia; only 53 (13.8%) of mothers said that SSC increases ‘Mother-Infant’ bonding.

#### Mothers’ reasons for not practicing SSC to their newborns

Mothers who didn’t practice SSC to their indexed infants also responded to a question about their reasons not to practice SSC to their newborns. About 126 (32.8%) of them replied that it was due to embarrassment (Additional file 3).

#### Factors associated with mothers SSC practice to their newborns

Binary logistic regression model showed that educational status, Occupation, Type of current pregnancy and numbers of ANC visits of mothers were associated with SSC practice of mothers to their newborns. Also, Mothers whose current pregnancy is unplanned, mothers who had attended ANC visits, gestational age of current pregnancy of mothers, mothers’ current birth attendant by health officer were significantly associated with SSC practice to their newborns (Table 3).

**Table 3:** Bivariate and multivariable logistic regression analysis of mothers SSC practice and its determinant factors during postpartum stay at public health institutions in four selected towns’ post natal units, Ethiopia, 2016/17

Variables	SSC status (N = 384)				COR (95% CI)	P-value	AOR (95% CI)	P-value
	Yes (n = 81)		No (n = 150)					
	n	%	n	%				
Educational status								
Unable to read and write	4	5.5	69	94.5	13.03 (4.33, 39.23)	0.000	18.23 (5.26, 63.52)	0.000*
Primary (1–8)	25	21.0	84	79.0	2.84 (1.52, 5.32)		3.72 (1.62, 8.52)	
Secondary (9–12)	45	39.8	68	60.2	1.14 (0.64, 2.05)		1.59 (0.75, 3.39)	
Tertiary (Diploma and above)	34	43.0	45	57.0	1.00		1.00	
Occupation								
Housewife	73	28.0	18	72.0	1.00		1.00	
Employed	32	35.6	858	64.4	0.70 (0.42, 1.17)		0.43 (0.11, 1.66)	
Unemployed four	3	9.1	30	90.9	3.88 (1.15, 13.12)	0.029	67 (0.16, 2.83)	0.582
Type of current pregnancy								
Wanted/planned	99	31.4	216	68.6	1.00		1.00	
Unwanted/unplanned	9	13.0	60	87.0	3.06 (1.46, 6.40)	0.003	0.59 (0.24, 1.45)	0.251
Number of ANC visits								
One	1	5.9	16	94.1	1.00		1.00	
Two	7	21.9	25	78.1	19.87 (2.67, 147.62)		9.97 (1.21, 81.91)	
Three	15	22.4	52	77.6	9.09 (1.18, 69.69)	0.003	8.55 (1.05, 69.54)	0.032*
Four	84	36.2	148	63.8	2.03 (0.84, 4.88)		1.31 (0.49, 3.50)	
GA (gestational age)								
< 37 weeks	7	77.8	2	22.2	1.00		1.00	
37–42 weeks	101	26.9	274	73.1	9.49 (1.94, 46.47)	0.005	0.07 (0.01, 0.53)	*0*.010*
Current birth attendant								
Midwife	88	32.1	186	67.9	1.00		1.00	
Nurse	6	20.0	24	80.0	1.89 (0.75, 4.79)		0.20 (0.07, 0.53)	
Doctor	6	24.0	19	76.0	1.49 (0.58, 3.88)		0.22 (0.06, 0.89)	
Health officer	8	14.5	47	85.5	2.78 (1.26, 6.13)	0.011	0.28 (0.07, 1.09)	0.212*

*COR* crude odds ratio, *AOR* adjusted odds ratio, *CI* confidence interval

* Significant association with P-value < 0.05

As it was clearly shown on the multivariable logistic regression analysis, after controlling for the effects of confounding variables, only two variables were significantly associated with mothers SSC practice to their newborns; educational status (AOR = 18.23 [95% CI 5.26, 63.52]), and number of ANC visits (AOR = 8.55 [95% CI (1.05, 69.54)] were significantly associated with SSC practice of mothers to their newborns (Table 3).

### Discussion

It is well documented that continuous uninterrupted SSC, which lasts for at least an hour has been shown to improve the success of EBF, maintains body temperature normal, maintains heart rate, respiratory rate and blood pressure normal [10, 17].

The prevalence of SSC Practice amongst mothers–infants indexed during the postpartum stay in this study was 28.1%. This finding was low as compared to studies in high-income countries in which SSC practice rate ranges from 60 to 70%. The prevalence of SSC in this study was lower as compared to developed countries. The variation might be due to mothers’ poor educational status (50%), 44.3% of mothers didn’t know SSC practice merits, and mothers were also lack knowledge and skills (31%) on how to provide SSC in the present study.

In this study, from mothers who didn’t practice SSC to their indexed infants about 32.8% of them put their reasons for not providing SSC to their newborns due to discomfort and lack of SSC knowledge and skills (31%).

This result was in line with the study conducted in Canada and northern Midwest United States, which showed about 20–30% of mothers interrupted breast feeding to their newborns due to discomfort during the postpartum stay [16, 18]. The reason for this might be around 25% of mothers in this study experienced instrumental delivery during their delivery which might prevent to provide SSC to their newborns because of the pain they develop.

SSC practice of mothers to their newborns is associated with their educational status. Illiterate mothers were 18 times more to practice SSC to their infants relative to their counterparts with mothers attended tertiary education (AOR = 18.23 [95% CI 5.26, 63.52]). These results could be explained that since majority of mothers in this study were housewives (68%) and they pass all their time with their newborns and feed their breast milk while employed mothers were mostly unable to do this.

Numbers of ANC visits was significantly associated with mothers SSC practice to their newborns during the postpartum stay. Mothers who had attended ANC visits three times were 8 times more likely to provide SSC to their newborns as compared to those who had attended only their first visit (AOR = 8.55 [95% CI 1.05, 69.54]). This could be explained that when mothers attended more ANC visits, their awareness will increase (more than half (55.7%) of mothers have good knowledge on the purpose of SSC) and adherence to care for them and the newborn become more effective, this results low compliance, and finally leads mothers to provide SSC to their newborns.

### Conclusion

This study revealed that the proportion of mothers’ SSC practice for at least 1 h to their indexed newborns’ during the postpartum stay was better (28.1%) as compared to other settings.

## Limitations

The cross sectional nature of the present study prevents any causal inferences.

## Additional files

**Additional file 1.** Schematic representation of the sampling procedure for the study on assessment of SSC during the postpartum stay and its determinants among mothers at public health institutions in four selected cities post-natal units, Ethiopia, 2016/17.

**Additional file 2.** Proportion of mothers SSC practice status to their indexed newborns during postpartum stay at public health institutions in four selected towns’ post-natal units, Ethiopia, 2016/17.

**Additional file 3.** Mothers reasons for not providing SSC to their indexed newborns during postpartum stay at public health institutions in four selected towns’ post-natal units, Ethiopia, 2016/17.

## Abbreviations

ANC: ante natal care
BEmONC: basic emergency obstetric and newborn care
C/S: cesarean section
CI: confidence interval
EBF: exclusive breast feeding
EDHS: Ethiopian Demographic Heath Survey
EMA: Ethiopian Midwife Association
EPHA: Ethiopian Public Health Association
FMoH: Federal Ministry of Heath
GA: gestational age
HC: health center
Kg: kilo gram
MNCH: maternal, newborn and child health
SPHMMC: St. Paul’s Hospital Medical Millennium College
SPSS: statistical package for social science
SSC: skin to skin contact
SVD: spontaneous vaginal delivery
UNICEF: United Nations Children’s Fund
