# Supplementary material for: Assessment of skin-to-skin contact (SSC) during the postpartum stay and its determinant factors among mothers at public health institutions in Ethiopia
Source: BMC Res Notes. 2019 Mar 14;12:136. doi: 10.1186/s13104-019-4176-5 (PMC6417157; doi:10.1186/s13104-019-4176-5)
Supplement: Supplementary file 1 — Additional file 1. Schematic representation of the sampling procedure for the study on assessment of SSC during the postpartum stay and its determinants among mothers at public health institutions in four selected cities post-natal units, Ethiopia, 2016/17. [file 13104_2019_4176_MOESM1_ESM.docx]

**All health facilities with post-natal units in five selected study areas**

Addis Ababa

Nh = 1890

Bahir Dar

Nh = 1160

Pawi

Nh = 295

**185**

**113**

**29**

Shashemene

Nh = 580

**57**

**Additional File 1:** Schematic representation of the sampling procedure for the study on assessment of SSC during the postpartum stay and its determinants among mothers at public health institutions in four selected cities post-natal units, Ethiopia, 2016/17.
