# Supplementary material for: Assessment of skin-to-skin contact (SSC) during the postpartum stay and its determinant factors among mothers at public health institutions in Ethiopia
Source: BMC Res Notes. 2019 Mar 14;12:136. doi: 10.1186/s13104-019-4176-5 (PMC6417157; doi:10.1186/s13104-019-4176-5)
Supplement: Supplementary file 2 — Additional file 2. Proportion of mothers SSC practice status to their indexed newborns during postpartum stay at public health institutions in four selected towns’ post-natal units, Ethiopia, 2016/17. [file 13104_2019_4176_MOESM2_ESM.docx]

**Additional file 2:** Proportion of mothers SSC practice status to their indexed newborns during postpartum stay at public health institutions in four selected towns’ post-natal units, Ethiopia, 2016/17.
